# In Situ Local Oxidation of SnO Induced by Laser Irradiation: A Stability Study

**DOI:** 10.3390/nano11040976

**Published:** 2021-04-10

**Authors:** Antonio Vázquez-López, David Maestre, Julio Ramírez-Castellanos, Ana Cremades

**Affiliations:** 1 Departamento de Física de Materiales, Facultad de CC. Físicas, Universidad Complutense de Madrid, 28040 Madrid, Spain; davidmaestre@fis.ucm.es (D.M.); cremades@fis.ucm.es (A.C.); 2 Departamento de Química Inorgánica, Facultad de CC. Químicas, Universidad Complutense de Madrid, 28040 Madrid, Spain; jrcastel@quim.ucm.es

**Keywords:** tin oxide, SnO, laser irradiation, phase transition, X-ray diffraction, romarchite

## Abstract

In this work, semiconductor tin oxide (II) (SnO) nanoparticles and plates were synthesized at room conditions via a hydrolysis procedure. X-ray diffraction (XRD) and transmission electron microscopy (TEM) confirmed the high crystallinity of the as-synthesized romarchite SnO nanoparticles with dimensions ranging from 5 to 16 nm. The stability of the initial SnO and the controlled oxidation to SnO_2_ was studied based on either thermal treatments or controlled laser irradiation using a UV and a red laser in a confocal microscope. Thermal treatments induced the oxidation from SnO to SnO_2_ without formation of intermediate SnO_x_, as confirmed by thermodiffraction measurements, while by using UV or red laser irradiation the transition from SnO to SnO_2_ was controlled, assisted by formation of intermediate Sn_3_O_4_, as confirmed by Raman spectroscopy. Photoluminescence and Raman spectroscopy as a function of the laser excitation source, the laser power density, and the irradiation duration were analyzed in order to gain insights in the formation of SnO_2_ from SnO. Finally, a tailored spatial SnO/SnO_2_ micropatterning was achieved by controlled laser irradiation with potential applicability in optoelectronics and sensing devices.

## 1. Introduction

Tin oxide is a well-known semiconductor oxide that commonly appears in two crystalline forms, tin dioxide (SnO_2_, cassiterite) and tin monoxide (SnO, romarchite) [1], the former being most commonly used because it is the stable polymorphic form. Despite the potential applicability of SnO in some fields of research, its use is commonly hindered as it is easily oxidized to the most stable SnO_2_, which is indeed one of the most extensively used semiconducting oxides.

Tin dioxide (tin oxide II, stannic oxide) (SnO_2_, Sn^4+^) cassiterite possesses n-type electronic properties. Its space group corresponds to rutile structural type, tetragonal P4_2_/mnm (136), crystalline structure with lattice parameters a = b = 4.738 Å and c = 3.186 Å [2], and it presents a wide band gap (E_G_~3.6 eV) at room temperature [3]. This versatile semiconductor oxide is commonly used in diverse fields, such as in gas sensors, catalysts, transparent semiconductors in solar cells, and as active material in anodes for ion-Li batteries, among others [2,4,5,6].

On the other hand, tin monoxide, (tin oxide II, stannous oxide) (SnO, Sn^2+^) romarchite, possesses p-type conductivity and often exhibits a layered structure with tetragonal space group P4/nmn (129) with lattice parameters a = b = 3.803 Å and c = 4.838 Å, corresponding to a litharge structure with a variable optical bandgap, ranging between E_G_ ~2.5–3.4 eV [1,3,7,8]. In recent years, SnO has generated increasing interest in photocatalysis [9,10] and as a potential thermoelectric material, supported by first principle calculations [11,12] in part due to its low toxicity and abundance compared with other thermoelectric materials. However, its use is still under-explored due to limitations concerning its synthesis in a pristine form without other Sn-based oxides and its easy oxidation to SnO_2_. Different synthesis routes have been employed up to now for the synthesis of SnO nanoparticles, such as the hydrothermal method [7,10,13] and microwave-assisted synthesis [14], whereas other methods such co-precipitation and hydrolysis remain less explored so far.

Despite SnO_2_ being considered a potential candidate for conductive material in ion-Li battery anodes [2], some authors such as Gervillié et al. [15] affirm that SnO appears to be a better candidate with regard to its irreversible capacities and coulombic efficiency, whereas SnO_2_ possesses the best gravimetric capacity. Moreover, because of its layered structure with sizeable c size, the use of SnO can overcome one of the main drawbacks in Li-ion batteries, the lattice expansion, leading as well to improved Li diffusion [16] or Na diffusion [17]. Furthermore, as one of the few p-type oxides, SnO is also considered as a candidate for the hole injection layer in optoelectronic devices. However, it is unlikely to be used as a high efficiency performing device if the SnO stability is not constant over time, this being one of the main drawbacks for the widening of SnO-based applicability. SnO naturally tends to oxidize to its allotropic and most stable SnO_2_ tetragonal rutile phase. Actually, in most synthesis methods, a mix of both tin oxides is formed, while the achievement of pure SnO is not straightforward. Hence, understanding the thermodynamics and limits of existence of this phase under different conditions (atmosphere, temperature, UV irradiation) is fundamental in order to overcome some of the challenges that face the applicability of SnO, which could then broaden the potential use of this oxide.

Moreover, the combination of n-type SnO_2_ and p-type SnO to form heterojunctions has also been gaining increasing attention in recent years, especially in optoelectronics, Li storage [18], and as chemiresistive sensor with enhanced sensibility for a variety of gases, such as NO_2_ [19], H_2_ [20], acetone [21], or formaldehyde gas [22]. In those cases, the variability of SnO geometries and hierarchical structures are fundamental to the optimal performance of the devices, while the achievement of a controlled local oxidation from SnO to SnO_2_ can also lead to the fabrication of p-n heterojunctions at the micro- and nanoscale with improved performance.

The creation of such SnO_2_/SnO structures can be complex because SnO is not stable, which may cause oxidation and diffusion processes between SnO and SnO_2_. Laser-assisted processing has arisen as an alternative method to thermal treatment for tailoring metal oxide semiconductors (MOs) that have been used in gas sensors, photocatalysts, solar cells, or thermistors [23]. Several parameters, such the laser intensity, pulse, or scanning rate, can be optimized to obtain the desired and controlled oxidation.

Herein, we report a combined study on both the stability under temperature and laser irradiation of as-synthesized SnO via hydrolysis, as well as the achievement of SnO_2_/SnO micropatterning via controlled laser irradiation. After the initial synthesis, nanoparticles were stored in containers under room conditions for several months. A complete study using transmission electron microscopy (TEM), X-ray diffraction (XRD), and Raman spectroscopy was performed. In order to study in detail the stability of the SnO oxidation to SnO_2_, thermo-XRD and controlled irradiation with a red or UV laser were performed. In this last case, a clear patterning was generated on the samples, which opens the field to micropatterning of complex SnO/SnO_2_ structures.

## 2. Materials and Methods

### 2.1. Synthesis of SnO Nanoparticles and Nanostructures

SnO nanostructures were synthesized following a soft chemistry route based on hydrolysis [2,8], carried out at room conditions, in contraposition with previous studies where Ar atmospheres were used [3]. Initially, the selected precursor SnCl_2_·2H_2_O (Sigma-Aldrich purity 99.99%, Darmstadt, Germany) was dissolved in water with continuous stirring at low temperature. Next, NH_4_OH (Sigma-Aldrich, Darmstadt, Germany) was added until pH = 8 was reached and hydrolysis occurred. Then, the temperature was raised to 100 °C for 2 h. The final product was centrifuged and washed several times until obtaining neutral pH; finally, it was dried at 50 °C for 12 h. The product was stored in glass vessels.

### 2.2. Characterization Techniques

The structural characterization of the nanoparticles was carried out by X-ray diffraction (XRD) in PANanalytical X’Pert Powder equipment (PANanalytical, Malvern, United Kingdom) using the copper Kα line λ_Cu_ = 1.5404 Å. Thermo diffractograms were performed with a X’Celerator detector in the range of 23.009° to 34.977°, with a step of 0.017° at controlled temperatures in the range of 25–900 °C, in steps of 50 °C between 50–200 °C (8 min rise, 4 min step), and in steps of 20 °C (4 min rise, 4 min step) between 200–800 °C, returning to steps of 50 °C from 800–900 °C. The microstructural analysis was carried out in a transmission electron microscope, by analyzing TEM images as well as selected area (electron) diffraction patterns (SAED/TEM) on a JEOL JEM 1400 plus (Jeol, Japan). Raman spectroscopy measurements were carried out at room temperature on a Horiba Jobin-Yvon LabRam Hr800 (Horiba, Kyoto, Japan) using both continuous wave He-Ne laser (λ = 633 nm) and He-Cd laser (λ = 325 nm). Different neutral filters were used to attenuate the total laser intensity, when necessary, diminishing the laser intensity from the nominal 5 mW or 13 mW (I_0_), respectively, for the UV or red laser to approximately 0.5·I_0_ (D03)_,_ 0.25·I_0_ (D06)_,_ 0.1·I_0_ (D1), or 0.01·I_0_ (D2) with the use of neutral filters [24]. The laser was focused onto the sample surface using a 40× objective (numerical aperture = 0.5, Thorlabs LMU-40X-NUV), which led to a laser spot diameter around 1 μm for the UV laser and a few microns for the red laser. The scattered light was collected with the same objective and dispersed with a grating of 2400 L/mm for UV and 600 L/mm for VIS and finally acquired with an air-cooled CCD detector Synapse. Photoluminescence (PL) was studied at room temperature in the same confocal microscope with a He-Cd UV laser (λ = 325 nm) as excitation source. The grating used for PL luminescence was 600 L/mm, using the same objective and CCD detector.

## 3. Results

### 3.1. TEM and XRD

XRD results confirmed that the synthesized nanoparticles mainly consisted of high crystalline romarchite SnO, with a dominant (101) peak. Only a weak maximum corresponding to SnO_2_ (110) was observed at 26.5° in the XRD diffractograms due to natural oxidation, while diffraction peaks related to metallic Sn or other Sn-based oxides were not detected in this case. The as-synthesized SnO was stable under room conditions. Figure S1 shows the XRD patterns acquired from SnO as synthesized and after 1 or 2 years of storage, when peaks from SnO still dominated the diffractograms after long storage and only a weak maximum from SnO_2_ was observed at 26.5°. In that time span, the weak contribution corresponding to SnO (001) was quenched, which may have been related to the formation of small domains of SnO_2_.

The lattice parameters a = 3.80 Å and c = 4.83 Å were estimated from the analysis of the corresponding XRD patterns using the SnO planes (101) and (002), as indicated in Table 1.

**Figure 1:**
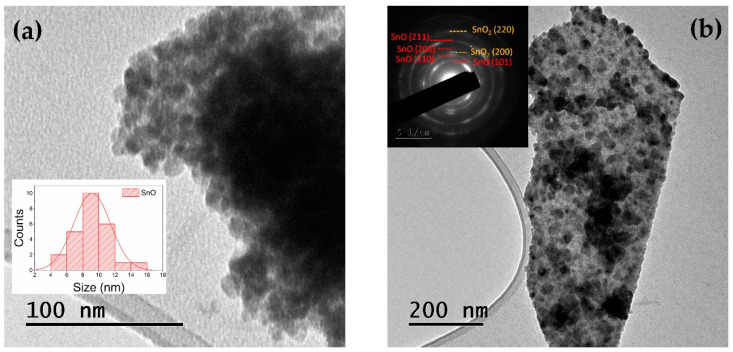
TEM image of the as-synthesized SnO nanopowder, showing (**a**) nanoparticles and (**b**) plate-like structures. Inset in (**a**) shows the histogram with the average dimensions, while inset in (**b**) shows the corresponding selected area electron diffraction (SAED) pattern.

TEM observations (Figure 1a) confirmed the presence of nanoparticles and plates, as described in a previous work [8]. Jaśkaniec et al. [16] recently suggested that solvent polarity on the synthesis method is strongly related to these 2D morphology changes. As an example, SnO can be obtained as platelets [8,17], rose-like particles [13,25,26], and other hierarchical architectures [9,10]. In our case, both plates and nanoparticles morphologies were formed via hydrolysis. 

From the low magnification TEM images analysis, it could be observed that the dimensions of the nanoparticles ranged from 5 to 16 nm, with averaged dimensions around 9 nm, as confirmed in Figure 1a. In addition, larger plates with dimensions of hundreds of nm could be also observed (Figure 1b). The SAED pattern included in the inset in Figure 1b confirmed that the nanocrystals corresponded to SnO, although a low amount of SnO_2_ was also observed in some regions, as confirmed by the weak spots in the SAED pattern, in agreement with the XRD results.

### 3.2. Thermo XRD

**Figure 2:**
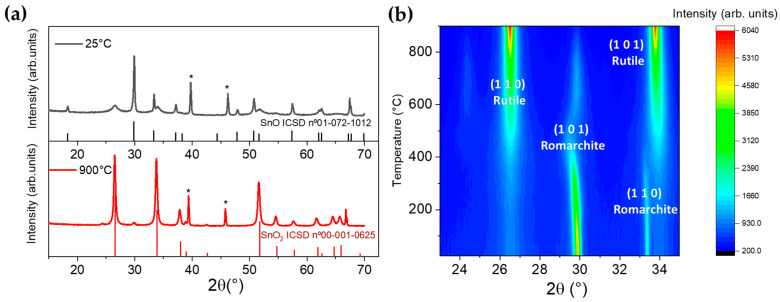
(**a**) XRD diffraction patterns of SnO powders at 25 and 900 °C and (**b**) thermo XRD pattern of SnO in the range of 23 to 35° represented in a contouring plot of intensities. Histogram patterns correspond to the Inorganic Crystal Structure Database (ICSD) files labelled in the figure. Peaks at 39.4 and 45.8° (marked with * in (**a**)) correspond to the Pt sample-holder.

To analyze the temperature stability of SnO and its oxidation to SnO_2_, XRD patterns were acquired *in situ* during a controlled annealing process in air, as described in the Materials and Methods section. Figure 2a shows the diffraction patterns acquired in the range of angles 15–70° at two temperatures, 25 °C and 900 °C. 

Figure 2b represents the contouring plot of the intensities from the XRD signal between 23–35° corresponding to the different steps in the annealing process from 25 to 900 °C. A detailed graph with the diffractograms recorded at temperatures between 25 and 900 °C is shown in Figure S2.

Peaks in the analyzed diffractograms corresponded only to SnO or SnO_2_ as they can be indexed according to the Inorganic Crystal Structure Database (ICSD) files nº 01-072-1012 for SnO and nº 00-001-0625 for SnO_2_, respectively. Diffraction peaks from metallic Sn or other oxides such as Sn_2_O_3_ and Sn_3_O_4_ were not detected in the analyzed range for the annealing temperatures. Peaks at 39.4 and 45.8°, marked with * in Figure 2a, correspond to the Pt sample-holder. It could be observed that oxidation from the initial SnO nanopowder into SnO_2_ started at around 400 °C. This oxidation process was nearly completed at temperatures above 800 °C, as observed in Figure 2b. Diverse authors also reported the oxidation from SnO to SnO_2_ at temperatures in the range 400–700 °C, as a function of the annealing parameters and the characteristic of the initial SnO nanopowder [13,14]. In general, annealing at temperatures higher than 600 °C fully transforms the SnO nanoparticles into the rutile SnO_2_ phase [27]. Moreover, in some cases an oxidation process involving the formation of intermediate oxides such as Sn_2_O_3_ and Sn_3_O_4_ has been reported [28]. In the present work, based on the thermodiffraction measurements, a direct oxidation process from unstable SnO to the more stable SnO_2_ phase occurred, probably by nucleation and growth process, without forming intermediate oxides. The thermodynamic phase diagram of the Sn-O system can be found in [1,29,30], which is in agreement with this work. 

Based on the results depicted in Figure 2b, it was observed that when increasing the temperature up to 300 °C there was no strong oxidation or phase transition and, in addition to a weak SnO_2_ (110) maximum at around 26.5°, only minor shifts to lower angles in the XRD peaks from SnO were observed, mainly the peak corresponding to the SnO (101) planes. Actually, the (110) peak from SnO_2_ rutile appeared and became as significant as the (101) maximum from SnO romarchite around 400 °C [27].

The (101) maximum from SnO observed at 29.9°, which dominated the XRD pattern at room temperature, suffered a shift to lower angles as the temperature increased. At 480 °C this peak was placed at 29.4° and then it shifted again toward higher angles, finally reaching 29.9° at 800 °C, although at this high temperature the relative intensity of this peak was drastically decreased. The shift to lower angles could be related to a thermal lattice expansion up to 480 °C, where the SnO_2_ phase dominated the XRD pattern. During the annealing and oxidation process an arrangement of the ions in the SnO lattice may have taken place, leading to small variations in the lattice constants along to the formation of SnO_2_. In this regard, the XRD maximum which exhibits more variation in its position and intensity corresponds to the (101) planes in romarchite. F. Wang et al. [31] reported a process in which the chemical coordination of the interstitials Sn cations becomes more similar to that of cassiterite SnO_2_ during the oxidation process, which could be related to the SnO (101) shift during annealing.

### 3.3. Raman Spectroscopy

In order to study the stability of SnO under irradiation and the possible laser-induced formation of SnO_2_ from SnO, Raman spectra from SnO nanopowder were analyzed using either a red (λ = 633 nm) or a UV (λ = 325 nm) laser, as well as variable laser intensity based on the employed neutral filters and controlled irradiation exposure time. Tin dioxide (SnO_2_) presented two active IR modes (A_2u_ and E_u_), four Raman active modes (A_1g_, B_1g_, B_2g_, and E_g_), and two inactive modes (A_2g_ and B_1u_) [32]. It is commonly reported that E_g_ (490 cm^−1^), A_1g_ (640 cm^−1^), and B_2g_ (760 cm^−1^) modes dominate the SnO_2_ Raman spectra. These modes are associated with the movement of O anions along the c-axis (E_g_) and elongation of O−Sn−O and movement of the anions in a symmetric (A_1g_) and asymmetric manner (B_2g_) orthogonal to the c-axis [2].

SnO, often shows two main Raman modes at around 110 and 208 cm^−1^ which correspond to the B_1g_ and A_1g_ modes, respectively [8,25]. However, there was still some controversy in the assignment of the Raman modes, as some authors assign the first peak to E_g_ based on frozen-phonon DFT calculations [33].

**Figure 3:**
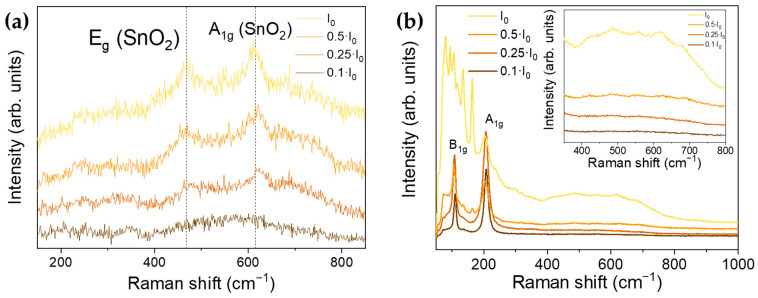
Raman spectra of the SnO sample with different energy density irradiations obtained with (**a**) UV laser (λ = 325 nm) or a (**b**) red laser (λ = 633 nm). Inset in (**b**) shows zoomed the region between 375–800 cm^−1^.

Figure 3a shows Raman spectra acquired with the UV laser in different spots on the SnO sample using different neutral filters, which can tailor the laser power densities, and using irradiation times of 60 s. Raman peaks from romarchite SnO were not observed when irradiating with the UV laser, even with the lowest power density (0.1·I_0_), at least within the resolution of the technique. As the laser power density increases, the Raman signal obtained with the UV laser is dominated only by vibrational modes from SnO_2_, the intensity of which rise, showing improved crystallinity of the formed SnO_2_. After the UV irradiation, the E_g_ and A_1g_ modes from SnO_2_ were clearly observed in the Raman spectra, but the former was slightly displaced to lower wavenumbers 468 cm^−1^, probably due to the initial lower crystallinity of the formed SnO_2_ or due to temperature shift of the phonon modes. As the irradiation density and duration increased, not only did the relative intensity of the Raman modes increase but also their positions were closer to the expected for SnO_2_. This formation of SnO_2_ from SnO by laser irradiation was irreversible, as expected, due to the higher stability of SnO_2_. Figure S3 shows Raman spectra acquired using the same filter but with variable irradiation time (60 or 600 s) where peaks from SnO_2_ increased for longer irradiation.

As irradiation with a UV laser induces the oxidation from SnO to SnO_2_, a red laser was also employed to achieve a deeper understanding of the intermediate steps in the transition from romarchite SnO to cassiterite SnO_2_. Raman spectra acquired with the red laser (λ = 633 nm) and using diverse neutral filters to modify the laser power density are shown in Figure 3b. An irradiation time of 200 s was used for the spectra acquisition. In this case, when using the lowest laser power density (0.1·I_0_), the formation of SnO_2_ was avoided, as only Raman peaks from SnO centered at 110.5 cm^−1^ and 208.6 cm^−1^ [34] could be clearly distinguished in the corresponding Raman spectrum. However, when using higher laser power densities, in addition to these SnO vibrational modes, some other new Raman peaks could be observed, mainly in the range 100–200 cm^−1^. Two main peaks centered at 136 and 165 cm^−1^ were clearly distinguished either using higher power intensity (I_0_) or increasing exposition time with lower power intensity (0.5·I_0_). Guillén et al. [35] attribute these peaks to intermediate SnO_x_ oxides, mainly to monoclinic Sn_3_O_4_, as also confirmed by other authors [36]. These results point out an intermediate oxidation from SnO to SnO_2_ by the formation of intermediate Sn_3_O_4_ when the red laser power density was not high enough. In addition, when the red laser power was maximum (I_0_), formation of SnO_2_ was promoted as wide modes appeared in the region between 450–700 cm^−1^ (inset in Figure 3b), which could be attributed to the E_g_ and A_1g_ modes from SnO_2_, also observed by UV irradiation (Figure 3a), although with much lower intensity in this case. It should be noted that contrary to the direct formation of SnO_2_ from SnO by thermal annealing, confirmed by XRD analysis, in this case either complete transition to SnO_2_ or formation of intermediate SnO_x_ phases could be also promoted as a function of the laser irradiation conditions.

### 3.4. Laser-Induced Phase Transition

**Figure 4:**
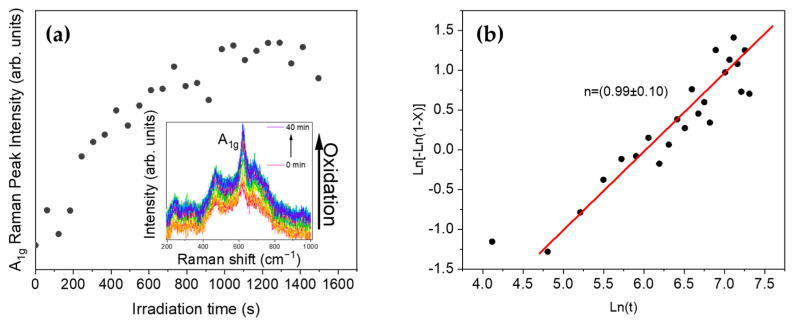
(**a**) Evolution of the A_1g_ Raman peak corresponding to SnO_2_ with increasing irradiation time, using the UV laser and the D03 filter (0.5·I_0_). Inset in (**a**) shows the spectra acquired using UV laser irradiation from 0 to 40 min. (**b**) Avrami plot from the data presented in (**a**).

Increasing UV laser irradiation was shown to induce a phase transition from SnO to SnO_2_ with minimum exposure time. Increasing this time enhanced the oxidation process and promoted SnO_2_ formation. SnO nanopowder was irradiated continuously with a UV laser using a power density of 0.5·I_0_, while Raman spectra were acquired in the same point each minute for 40 minutes. As observed in the inset in Figure 4a continuous laser irradiation led to higher intensity spectra and narrow and well-centered peaks corresponding to the SnO_2_ modes, such as A_1g_. Peak intensities as a function of the irradiation duration in Figure 4a could be fitted to the Johnson–Mehl–Avrami–Kolmogorov (JMAK) equation [37]:

*X* = 1 − exp(−*K*t^*n*^)

where, *X* is referred to the volume fraction that is transformed in the irradiation zone, *K* is the effective rate constant, and *n* is the Avrami exponent. This exponent depends on the mechanism of nucleation and growth. In this case we considered that the volume transformed was proportional to the measured Raman intensity obtained by subtraction of a linear profile background.

The JMAK equation has often been employed to describe laser-induced phase transitions from metastable phases [24] or to describe crystallization kinetics. In this case the transformation from SnO to SnO_2_ could not be described with this equation as the transformation for high laser energy densities occurred very fast. However, evolution and crystallization of the SnO_2_ formed during the initial irradiation stages could be described with the fit depicted in Figure 4b. In this case, the calculated Avrami exponent had a value close to n = 1, which corresponds to a diffusion controlled bi-dimensional reaction with nucleation site saturation. Hence, increasing irradiation time enhanced SnO_2_ crystallinity

**Figure 5:**
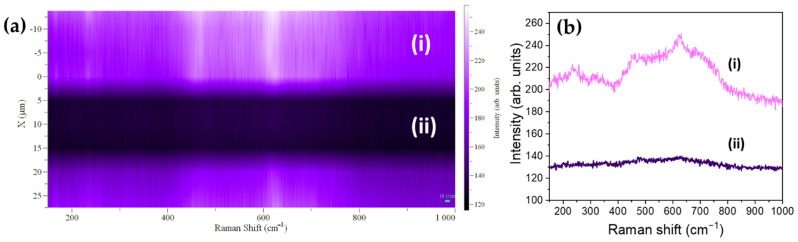
(**a**) Line patterning induced by UV irradiation. Two clear different zones can be observed corresponding to (i) SnO_2_ obtained after UV irradiation and (ii) SnO in the non-irradiated region. (**b**) Raman spectra acquired with the UV laser on regions (i) and (ii). Mapping in (**a**) was obtained by scanning both regions (irradiated and non-irradiated) with 0.1·I_0_.

Advantage can be taken from the laser-induced oxidation from SnO to SnO_2_ in order to achieve spatial controlled SnO/SnO_2_ micropatterning. Using a monitored stage, the UV laser with the highest laser intensity I_0_ could irradiate different areas in the sample with micrometric resolution as a function of the laser spot size, thus leading to a spatial controlled formation of SnO_2_. Following this process, SnO or SnO_2_ regions could be promoted in the samples as a function of the selected patterning. In this case, Figure 5a shows the bright (SnO_2_) or dark (SnO) stripe-patterning formed on the surface of the sample after a controlled irradiation with the UV laser only in the bright areas. After the controlled UV irradiation, Raman spectra were acquired in selected points from the regions in Figure 5a using the UV laser and the lowest power density (0.1·I_0_) to avoid formation of SnO_2_ during measurements. The spectra acquired in region (i) corresponded with the Raman signal from SnO_2_, confirming oxidation from SnO to SnO_2_ only in the irradiated regions, as shown in Figure 5b. Therefore, the controlled UV laser irradiation could tailor the oxidation process leading to either n-type SnO_2_ and/or p-type SnO spatial distribution following a micrometric patterning. The availability of both n and p type conductivity in Sn oxides allows the potential development of bipolar devices based on p-n heterojunctions controlled at the microscale. Moreover, intermediate SnO_x_ oxides could be also formed if needed as a function of the irradiation conditions. 

### 3.5. Photoluminescence

**Figure 6:**
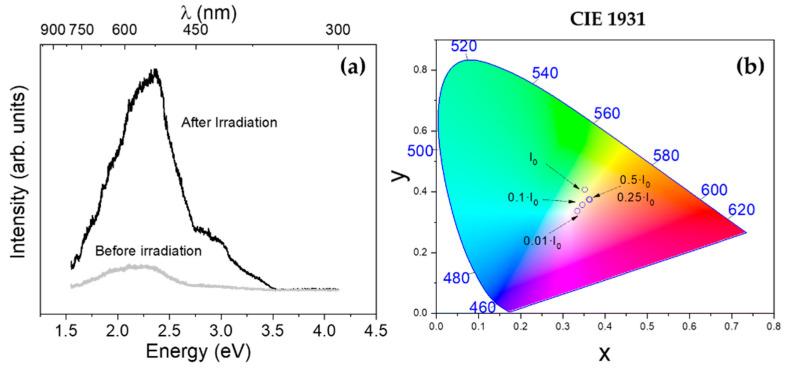
(**a**) PL of SnO nanoparticles before irradiation (acquired with a 0.1·I_0_ neutral filter) and after irradiation with I_0_ for 10 s (acquired with a 0.1·I_0_ neutral filter). (**b**) *Comission Internationale de l’Eclairage* (CIE) 1931 plot corresponding to PL intensity of SnO nanoparticles using different neutral filters.

SnO bandgap is commonly reported to be between 2.5 eV (~496 nm) and 3.4 eV (~364 nm), therefore, an UV laser of λ = 325 nm (~3.8 eV) was used as excitation source in order to get information from all the luminescent processes. The oxidation to SnO_2_ by UV irradiation took place very fast, as observed during Raman analysis; thus, the obtained photoluminescence signal resembled the characteristic luminescence spectrum from SnO_2_. In this case, neutral filters were also used to reduce the laser power intensity. Figure 6a shows PL spectra from SnO acquired with the UV laser using 0.1·I_o_ laser power density in the same spot, before and after irradiating the sample with the maximum (I_o_) intensity for 10 s. 

The total intensity of the PL signal was increased when using higher laser power densities, as expected. For the PL spectrum acquired with the highest laser intensity (I_0_), the dominant emission was centered at around 2.25 eV, as shown in Figure 6a. Deconvolution into Gaussian functions of the spectra shown in Figure 6a can be found in Figure S4. Additionally, a shoulder at about 3 eV up to 3.5 eV, could be observed. This spectrum can be attributed to SnO_2_, as this material normally shows characteristic emissions at 1.94 eV and 2.25 eV associated with oxygen vacancies-related defects: ~2.50 eV due to surface defect states [2,38] and ~3 eV due to transitions involving Vo’’ levels. 

Before the irradiation, the PL spectrum was characterized by two main emissions centered at around 2 and 2.3 eV. These emissions could be due to high defective SnO_2_, although the presence of SnO, not detected by UV Raman analysis, could not be completely disregarded, as some authors reported emissions from SnO around 2 to 2.3 eV due to defects such as Sn vacancies and O vacancies, and at ~3 eV related to band-edge emissions [8,39]. The differences on the PL spectra acquired using different filters is shown in Figure 6b, represented as chromaticity coordinates calculated from the PL spectra according to the *Comission Internationale de l’Eclairage* (CIE 1931) standard [40,41]. In any case, careful attention should be paid during analysis of discussion of the PL signal from SnO, as possible formation of SnO_2_ during irradiation should be considered. 

## 4. Conclusions

Crystalline SnO nanoparticles and plates were synthesized via a hydrolysis method that allowed achievement of a large amount of SnO nanopowder, avoiding an atmosphere-control during the synthesis. The nanoparticles dimensions ranged from 5 to 16 nm, as confirmed by TEM observations. XRD measurements confirmed the minor oxidation and stability of the as-synthesized SnO nanopowder stored at room conditions for up to 24 months. The oxidation from SnO to SnO_2_ was promoted by thermal annealing or by controlled laser irradiation. Temperatures above 300 °C were required to initiate the oxidation from SnO to SnO_2_ which was completed at 800 °C, following a nucleation and growth process without formation of intermediate SnO_x_, as confirmed by thermodiffraction measurements. On the contrary, by using UV or red laser irradiation the transition from SnO to SnO_2_ could be controlled, assisted by the formation of intermediate Sn_3_O_4_, as confirmed by Raman spectroscopy. Careful attention should be paid during the PL and Raman analysis, as the use of the laser as excitation source can induce formation of SnO_2_ during measurement, thus leading to possibly misleading results and discussion. In this work, the evolution from SnO to SnO_2_ was studied as a function of the laser excitation source (UV and red laser), the laser power density (controlled by using neutral filters), and the irradiation duration in order to achieve deeper knowledge and control of the oxidation process. The evolution of the oxidation of the SnO_2_ promoted by UV laser irradiation obeys Avrami behavior. Finally, advantage was taken from the controlled SnO oxidation and a tailored spatial SnO/SnO_2_ micropatterning was developed based on a controlled laser irradiation. This laser-induced micropatterning can be of potential interest for the fabrication of p-n devices based in all Sn-oxides with applicability in electronic, optoelectronic, and sensing devices.

## Figures and Tables

**Table 1:** Averaged dimensions and lattice parameters for tin oxide (SnO) estimated from TEM and XRD measurements.

D(nm) (By TEM)	a(Å) (By XRD)	c(Å) (By XRD)
9.14 ± 2.58	3.80(1)	4.83(7)

## Supplementary Materials

The following are available online at https://www.mdpi.com/article/10.3390/nano11040976/s1, Figure S1: XRD patterns on the period span of 2 years, Figure S2: (a) Complete set of thermodiffractograms and (b) detailed region between 29 and 30°, Figure S3: Raman spectra acquired using the same filter but with variable irradiation time (60 or 600 s), Figure S4: Photoluminescence deconvolution to Gaussian functions of SnO (a) before and (b) after irradiation.
