# Supplementary material for: In Situ Local Oxidation of SnO Induced by Laser Irradiation: A Stability Study
Source: Nanomaterials (Basel). 2021 Apr 10;11(4):976. doi: 10.3390/nano11040976 (PMC8070038; doi:10.3390/nano11040976)
Supplement: Supplementary file 1 [file nanomaterials-11-00976-s001.pdf]

Supplementary Materials

In situ local oxidation of SnO induced by laser irradiation: A stability study

Antonio Vázquez-López [1,*], David Maestre [1], Julio Ramírez-Castellanos [2], Ana Cremades [1]

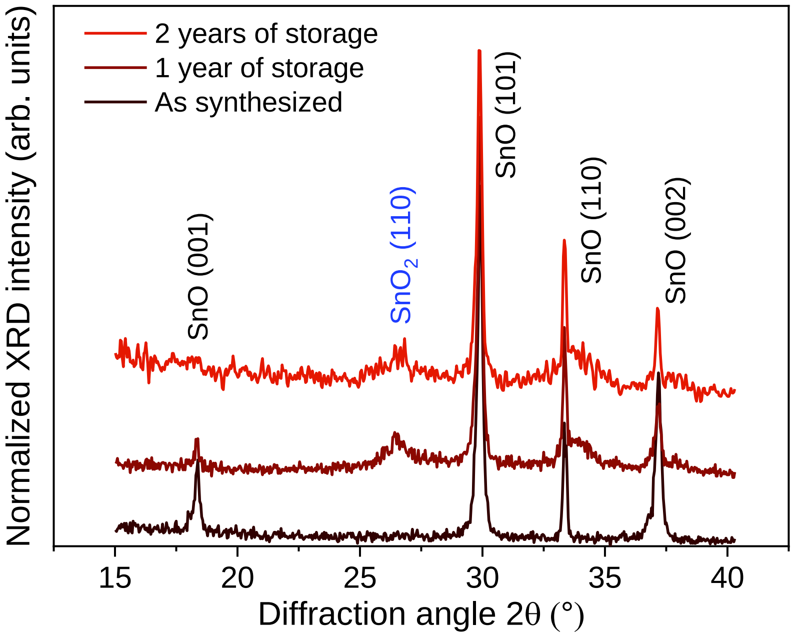

Figure S1. XRD patterns on the period span of 2 years

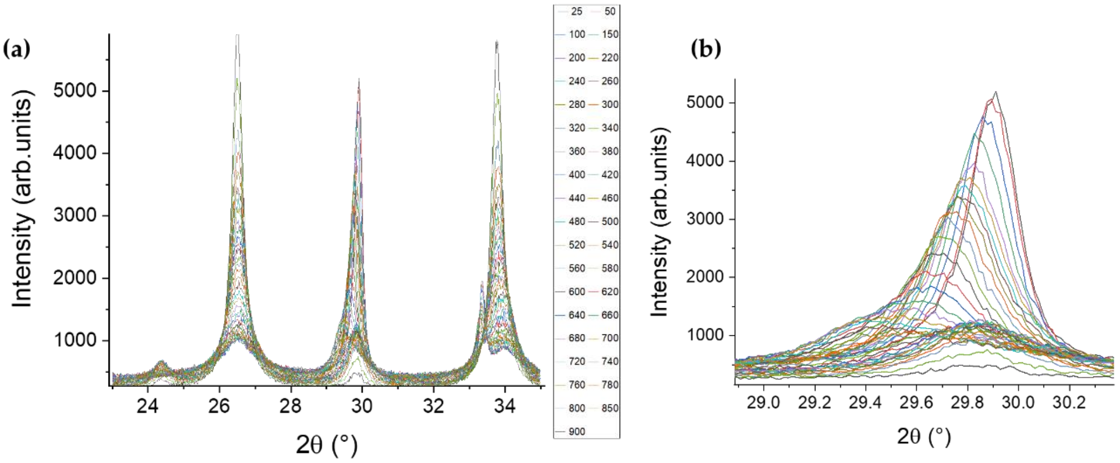

Figure S2. (**a**) Complete set of thermodiffractograms and (**b**) detailed region between 29 and 30°

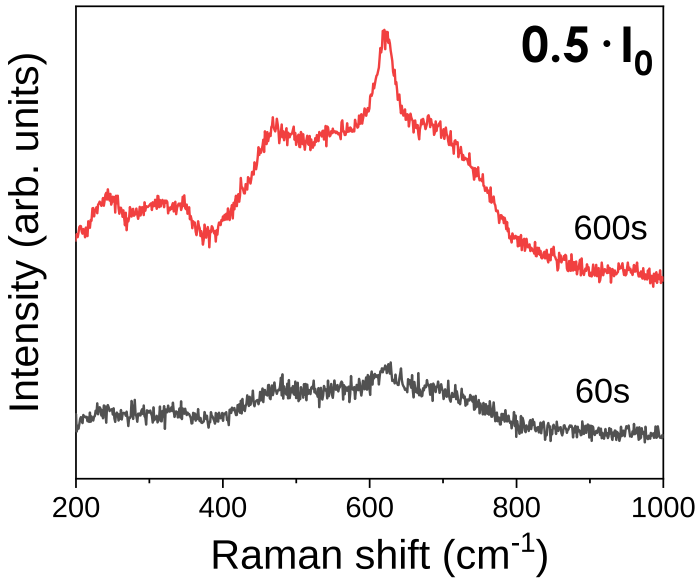

Figure S3. Raman spectra acquired using the same filter but with variable irradiation time (60 or 600s)

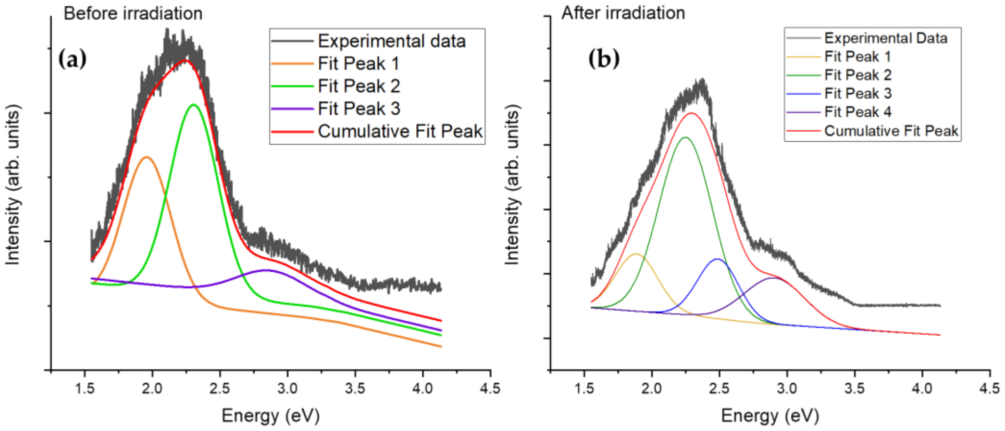

Figure S4. Photoluminescence deconvolution to Gaussian functions of SnO (**a**) before and (**b**) after irradiation.
